# Under-Recognized Melioidosis in Indonesia: Evidence of Diagnostic Misidentification in Bali

**DOI:** 10.3390/pathogens15080835

**Published:** 2026-08-10

**Authors:** Ni Nyoman S. Budayanti, Putu N. C. Santoso, Komang J. P. Pinatih, I Wayan A. G. M. Saputra, Robert Norton

**Affiliations:** 1 Faculty of Medicine, Udayana University, Denpasar 80113, Bali, Indonesia; 2 Faculty of Medicine and Health Sciences, Warmadewa University, Denpasar 80235, Bali, Indonesia; 3 Bali Mandara General Hospital, Denpasar 80235, Bali, Indonesia; 4 College of Medicine and Dentistry, James Cook University, Townsville, QLD 4814, Australia

**Keywords:** melioidosis, Bali, misidentification, *Burkholderia pseudomallei*

## Abstract

Melioidosis is rarely reported from Bali, Indonesia. This study aimed to determine whether *Burkholderia pseudomallei* was being misidentified. One hundred isolates that had been previously otherwise identified were screened using PCR targeting two specific genes and sequencing. Five were confirmed as *B. pseudomallei* and had been misidentified as *Pseudomonas aeruginosa*.

## 1. Introduction

*Burkholderia pseudomallei*, the causative agent of Melioidosis, is endemic, particularly in Southeast Asia and Northern Australia. Although Indonesia lies within this endemic belt, data likely underestimates the true burden of disease in this region. It has been estimated that Indonesia could experience more than 20,000 cases annually, with an estimated case fatality rate of 51%. This would potentially result in over 10,000 deaths per year [1]. Indonesia is categorized as a country where melioidosis is likely endemic but substantially underreported. Limited awareness among healthcare professionals contributes to this gap. Historically, eight reports of melioidosis in Indonesia were published prior to the 1960s, followed by a nearly five-decade absence of documented cases. In 2005, melioidosis was reported in four pediatric and adolescent tsunami survivors in Aceh, Indonesia, who were hospitalized with pneumonia [2]. Since then, additional cases have been documented in Java, Sulawesi, Sumatra, Kalimantan, and East Nusa Tenggara, with nearly half resulting in fatal outcomes [3]. In Bali Province, however, reported cases remain limited. The apparent absence of this organism in Bali has led to this study, which determines whether the absence is real or simply a result of laboratory misidentification.

Diagnostic challenges further contribute to under-detection. Limited familiarity with its colony morphology may lead to misidentification. Serological tests also demonstrate variable sensitivity and specificity, limiting their diagnostic reliability [4].

The present study aimed to determine whether *B. pseudomallei* is truly absent from clinical specimens in this setting. This will also determine whether misidentification of oxidase-positive Gram negative bacilli has led to missed diagnoses.

## 2. Materials and Methods

This study was conducted at the Rumah Sakit Umum Pusat (RSUP) Ngoerah, a 760-bed tertiary referral hospital located in Denpasar, Bali, Indonesia. The study employed a cross-sectional observational design. One hundred oxidase-positive Gram negative bacilli isolated in the Clinical Microbiology Laboratory of RSUP Ngoerah, Bali, were collected over a four-month period between March and June 2019. Isolates that were included were 100 sequential isolates of Gram negative bacilli that were oxidase-positive. These were collected over the four month trial period as previously mentioned. All other isolates were excluded. All collected isolates were stored at −80 °C until further analysis. Prior to molecular testing, isolates were subcultured on MacConkey agar. Isolates were confirmed to be Gram negative with a standard Gram’s stain. Oxidase positivity was determined by a manual oxidase test. All isolates had been previously put through the Vitek2 (bioMérieux, Marcy l’Étoile, France) system and been identified as organisms other than *B. pseudomallei*. Bacterial colonies were subsequently subjected to polymerase chain reaction (PCR) using primers specific for *B. pseudomallei*. Two primer sets were used in this study. The first targeted the 16s rRNA gene and if positive, a second PCR assay targeting the orf2 gene within the type III secretion system 1 (TTSS1) region was used (GenBank accession no. AF074878, positions 3934–4481), as previously described [5]. A positive control of *B. pseudomallei* was obtained from the University of North Sumatra and used in this study. The following reference sequences were used.

The 16S rRNA primer sequence that was used is listed below:

F: 5′-CTTTCGAGCACGCCCAACTCTCAT-3′.

R: 5′-CTTCGGCCTGGTGGCGAGTGGC-3′.

The second PCR used that targeted the orf2 gene within the TTSS1 gene had sequences as below.

F 5′-CGTCTCTATACTGTCGAGCAATCG-3′.

R 5′-CGTGCACACCGGTCAGTATC-3′.

All PCR products that showed successful amplification for both the orf2-specific primer set and the 16S rRNA gene target, and two samples that had positive amplification only for the 16S rRNA gene target, were chosen for sequencing to confirm bacterial identity. The PCR products of these isolates were then purified and sent to Genetica Science Indonesia (Jakarta, Indonesia), a commercial sequencing facility, for nucleotide sequence determination by Sanger sequencing. SnapGene (Boston, MA, USA) was used to open the sequencing result, and quality trimming was performed to ensure that only those sequences with acceptable chromatograms were used. The Molecular Evolutionary Genetics Analysis (Mega X version 12) system was used to align and complete the missing areas to obtain the final completed sequences from all of the samples. The final edited sequences were then entered into the NCBI Blast online tool to confirm the identity of the isolates.

The raw chromatogram files obtained from sequencing were first reviewed for quality and edited to correct any sequencing errors prior to downstream analysis. Next, the finalized nucleotide sequences were compared with reference sequences in the National Center for Biotechnology Information (NCBI) BLAST database https://blast.ncbi.nlm.nih.gov/Blast.cgi?PROGRAM=blastn&PAGE_TYPE=BlastSearch&LINK_LOC=blasthome, accessed on 1 June 2026, with the aim of verifying the identities of the bacterial isolates. Taxonomic assignment and confirmation of the PCR-based detection results were based primarily on sequence similarity and query coverage.

Prior identification data were retrieved from the initial automated testing performed using the VITEK^®^2 system (bioMérieux, Marcy l’Étoile, France). Clinical data of patients with culture and PCR-positive results were reviewed through medical records. Isolates with positive PCR results for either the 16S rRNA PCR assay or the TTSS1 orf2 gene assay were further subcultured on Ashdown agar, and colony morphology was observed daily for up to four days. Detailed morphological characteristics were recorded. This study was approved by the Ethics Committee of the Faculty of Medicine, Universitas Udayana (15 April 2019, Approval No. 117/UN14.2.2.VII.14/LP/2019).

## 3. Results

Of the 100 isolates, 9 produced distinct amplicons with the 16s rRNA target. A further 22 isolates were considered presumptively positive, as they exhibited either faint bands of an expected size or a clear band of an unexpected size from the 16s rRNA PCR. The remaining 69 isolates were negative, with no detectable amplification products observed.

**Figure 1:**
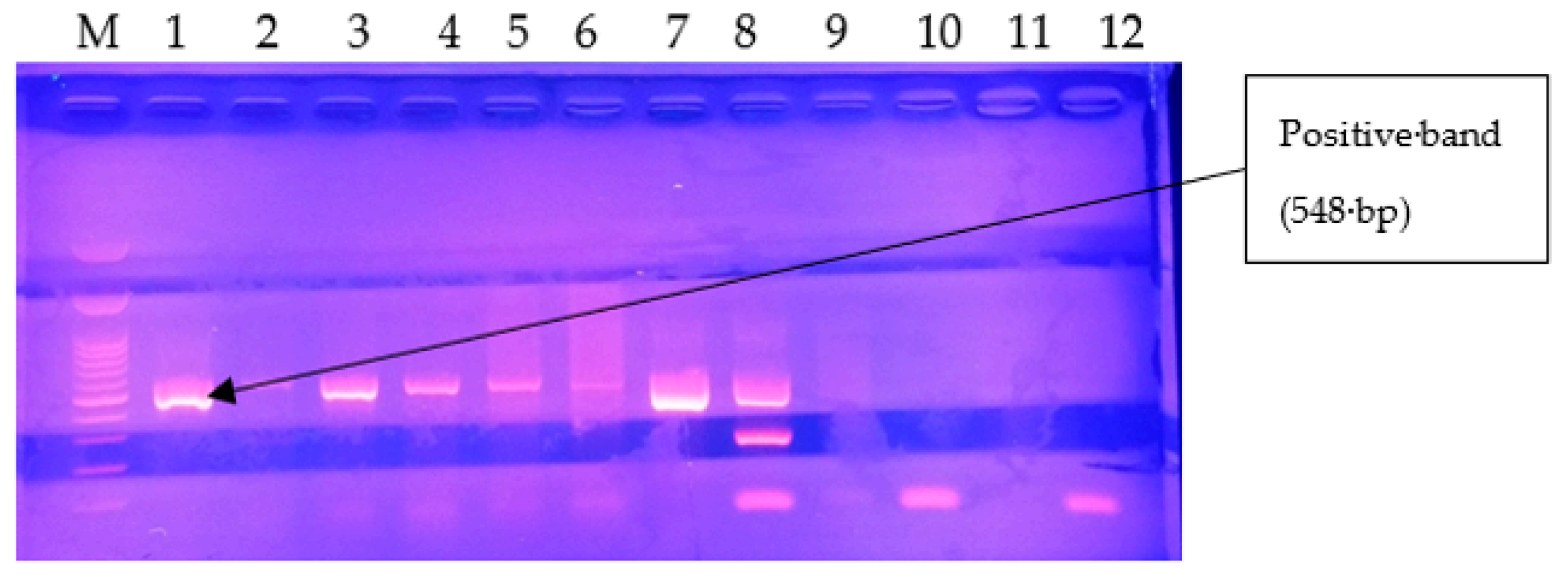
PCR amplification results of the TTSS1 orf2 gene among isolates previously positive for 16S rRNA PCR. From left to right: molecular weight marker (M); positive control (lane 1); isolate #62 (lane 2); isolate #63 (lane 3); isolate #64 (lane 4); isolate #67A (lane 5); isolate #67B (lane 6); isolate #67C (lane 7); isolate #68 (lane 8); isolate #75A (lane 9); isolate #75B (lane 10); isolate #92A (lane 11); and isolate #92B (lane 12).

**Figure 2:**
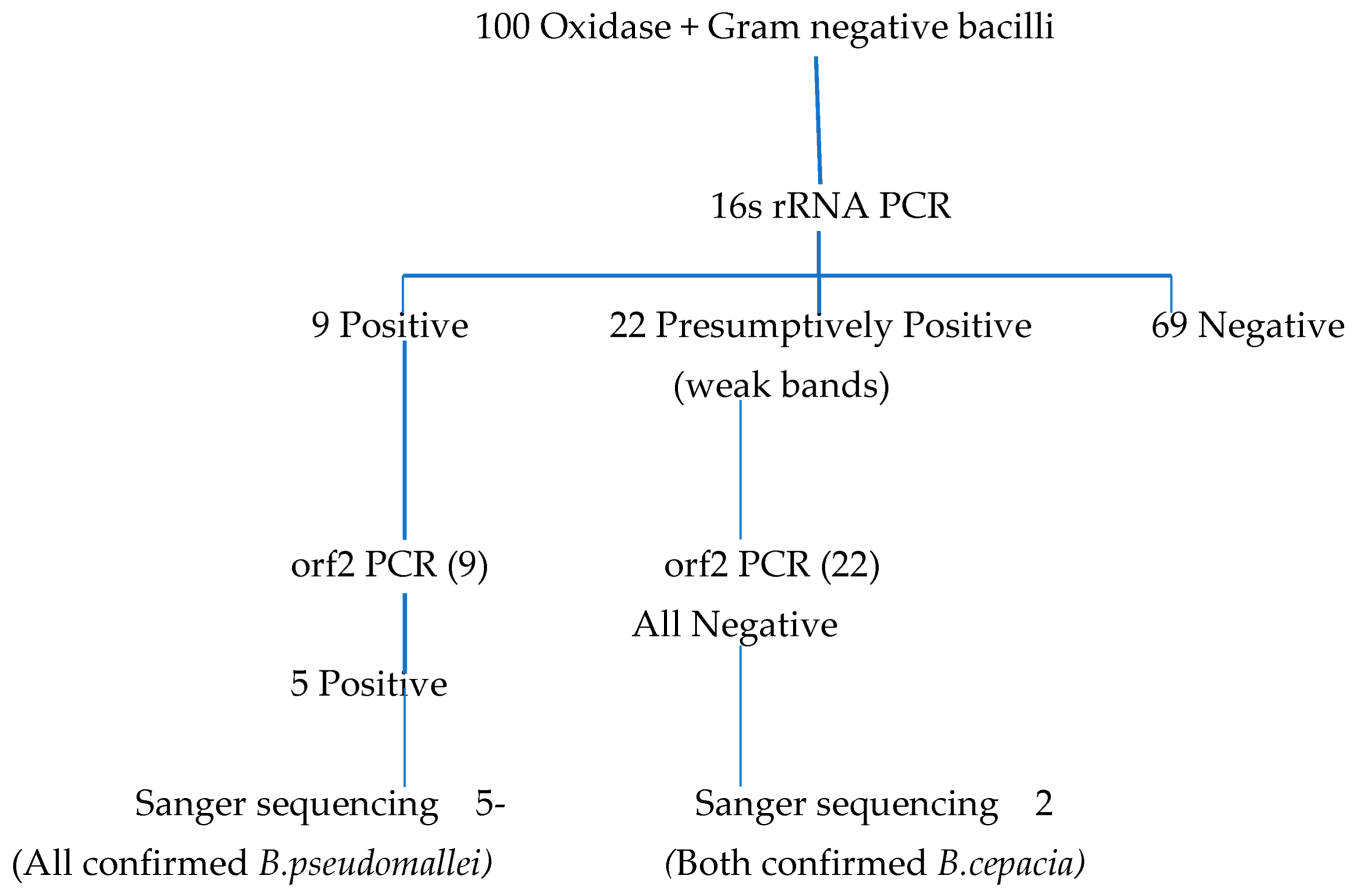
Flow chart summarizing the process of confirmation of identity as *B. pseudomallei* in 100 oxidase-positive, Gram negative bacilli.

All 31 isolates that yielded positive or presumptively positive results in the 16S rRNA PCR assay were subsequently subjected to a second PCR targeting the TTSS1 orf2 gene. Among the 22 isolates initially classified as presumptively positive, all were negative when tested with the orf2 primer set. Of the 9 isolates that demonstrated clear positive amplification with the 16S primers, only 5 produced the expected amplicon of 548 bp with the TTSS1 orf2 primers as shown in Figure 1. According to laboratory records, these isolates were initially identified as *Pseudomonas aeruginosa* using the VITEK^®^2 automated system (bioMérieux, Marcy l’Étoile, France). Of the 7 sequenced isolates, 5 were confirmed as *B. pseudomallei*. This is shown in Table 1. A flowchart showing the breakdown of the isolates tested is provided in Figure 2. Details on the sequencing chromatograms and alignments are provided in Supplementary Files (Figures S1–S4).

A retrospective clinical review was conducted using the medical records of the 5 patients from whom culture isolates were confirmed as *B. pseudomallei* by PCR and sequencing. Details are shown in Table 2. All 5 patients were male, with ages ranging from 6 to 71 years. All patients had recognized risk factors for melioidosis, including diabetes mellitus, malignancy, chronic kidney disease, and alcohol dependence. The 2 isolates that were not confirmed as *B. pseudomallei* were shown to be *Burkholderia* spp. Most patients did not receive appropriate antimicrobial therapy for melioidosis. Only 1 patient was treated with meropenem, administered late during hospitalization. The reason for the choice of meropenem is unclear. Three patients died, including the patient who had received meropenem.

## 4. Discussion

The 16s rRNA sequence of *B. pseudomallei* demonstrates a high degree of similarity to other species within the genus. This included *B. cepacia*, *B. gladioli*, *B. mallei*, *B. thailandensis*, and *B. ubonensis*. It has been reported that the amplified 16S rRNA regions of *B. cepacia*, *B. gladioli*, and *B. thailandensis* share up to 99.059% sequence similarity with *B. pseudomallei* and *B. mallei*, while *B. pseudomallei* and *B. mallei* differ by only a single nucleotide at position 75 [6]. Similarly, it has been demonstrated that the 16S rRNA sequences of *B. pseudomallei* and *B. thailandensis* differ by approximately 1%, underscoring the limited discriminatory power of 16S rRNA-based assays for definitive species-level identification [7].

The limited specificity of the 16S primer set may serve as a preliminary screening tool for *Burkholderia* spp., although it lacks sufficient specificity for definitive identification of *B. pseudomallei*.

By contrast, PCR targeting the orf2 gene within the Type III Secretion System 1 (TTSS1) demonstrated high specificity. The five isolates positive for orf2 were confirmed as *B. pseudomallei* by sequencing. Notably, all five isolates had been misidentified as *Pseudomonas aeruginosa*.

Phenotypic heterogeneity in *B. pseudomallei* is well recognized, making culture-based detection challenging [8]. In retrospect, using basic bench tests such as gentamicin and colistin resistance together with amoxicillin–clavulanate susceptibility might have facilitated correct preliminary identification [9]. It is, however, recognized that occasional isolates will exhibit gentamicin susceptibilility. These have been isolated in Sarawak [10]. The limitations of the VITEK^®^2 system (bioMérieux, Marcy-l’Étoile, France) in the identification of this organism have been described. A recent study found that the Vitek 2 system correctly identified only 16 of 23 (69.6%) known *B. pseudomallei* isolates [11].

All confirmed positive cultures were obtained in early May 2019. This corresponded to the late rainy season and transitional monsoon period in eastern Indonesia. This is epidemiologically consistent with exposure during the preceding period of intense rainfall during April 2019 [12].

All confirmed cases occurred in males, consistent with global epidemiological data indicating that 60–70% of melioidosis cases occur in men [13]. In this cohort, only one patient received meropenem, initiated late in the course of illness, and four patients received therapy that lacked optimal activity against *B. pseudomallei*. This likely contributed to the high mortality observed, underscoring the critical importance of accurate and timely diagnosis in endemic settings.

## 5. Conclusions

This study confirms the presence of *Burkholderia pseudomallei* in Bali and demonstrates that melioidosis is likely underrecognized due to phenotypic misidentification and limited clinical suspicion. Automated systems misidentified all confirmed isolates and delayed appropriate antimicrobial therapy. This was associated with high mortality. Screening with basic bench tests might assist in early diagnosis. The results from the use of automated biochemical identification systems, while cost-effective in the busy microbiology laboratory, need to be interpreted with caution. Implementation of specific molecular diagnostics, particularly TTSS1-targeted PCR, is suggested to improve correct identification, guide appropriate treatment, and reduce mortality in endemic settings such as Indonesia.

## Figures and Tables

**Table 1:** BLAST analysis results of sequencing products from isolates positive for the 16S rRNA and *orf2* (TTSS1) genes.

Isolate No	Primer Target	Phenotypic Identification by VITEK^®^2	NCBI BLAST Result
9	16S	*Burkholderia cepacia*	*Burkholderia* spp.
62	16S	*Pseudomonas aeruginosa*	*Burkholderia pseudomallei*
	*orf2*		*Burkholderia pseudomallei*
63	16S	*Pseudomonas aeruginosa*	*Burkholderia pseudomallei*
	*orf2*		*Burkholderia pseudomallei*
64	16S	*Pseudomonas aeruginosa*	*Burkholderia pseudomallei*
	*orf2*		*Burkholderia pseudomallei*
67	16S	*Pseudomonas aeruginosa*	*Burkholderia pseudomallei*
	*orf2*		*Burkholderia pseudomallei*
68	*orf2*	*Pseudomonas aeruginosa*	*Burkholderia pseudomallei*
92	*16S*	*Burkholderia cepacia*	*Burkholderia* spp.

**Table 2:** Clinical characteristics and outcomes of patients with culture and PCR confirmed *Burkholderia pseudomallei* infection.

Variable	Case #62	Case #63	Case #64	Case #67	Case #68
Age/Sex	71 years/Male	49 years/Male	6 years/Male	56 years/Male	21 years/Male
Date of admission	25 April 2019	26 April 2019	1 April 2019	6 May 2019	16 March 2019
Occupation	Trader	Farmer	Not available	Farmer	Not available
Residence	Tabanan, Bali	Southwest Sumba, East Nusa Tenggara	Timor Leste	Sikka, East Nusa Tenggara	Denpasar, Bali
Culture specimen	Pleural fluid	Wound swab	Blood	Blood	Wound swab
Provisional diagnosis	Hydropneumothorax	Post-colostomy	Acute leukemia	Urinary sepsis	Fractures of Maxilla and femur
Risk factors/comorbidities	Chronic kidney disease, HIV infection on antiretrovirals, benign prostatic hypertrophy	Carcinoma of the ascending colon	Not available	Benign prostatic hypertrophy, Diabetic	Trauma, alcohol
Outcome	Death	Death	Discharged	Death	Discharged

## Data Availability

The data presented in this study are available on request from the corresponding author.

## Supplementary Materials

The following supporting information can be downloaded at: https://www.mdpi.com/article/10.3390/pathogens15080835/s1, Figure S1. Chromatogram of the sequence result of 16S gene from sample #64; Figure S2. Alignment of the 16S sequencing result using MEGA X to complete the areas where the chromatogram quality was not good to obtain the final completed sequence of the 16S for the sample; Figure S3. Chromatogram of the sequence result of ORF2 gene from sample #64; Figure S4. Alignment of the orf2 sequencing result using MEGA X to complete the areas where the chromatogram quality was not good to obtain the final completed sequence of the orf2 for the sample.
