# Supplementary material for: Under-Recognized Melioidosis in Indonesia: Evidence of Diagnostic Misidentification in Bali
Source: Pathogens. 2026 Aug 10;15(8):835. doi: 10.3390/pathogens15080835 (PMC13516223; doi:10.3390/pathogens15080835)

Supplementary Figures (S1 -S4)

Figure S1. Chromatogram of the sequence result of 16S gene from sample #64.

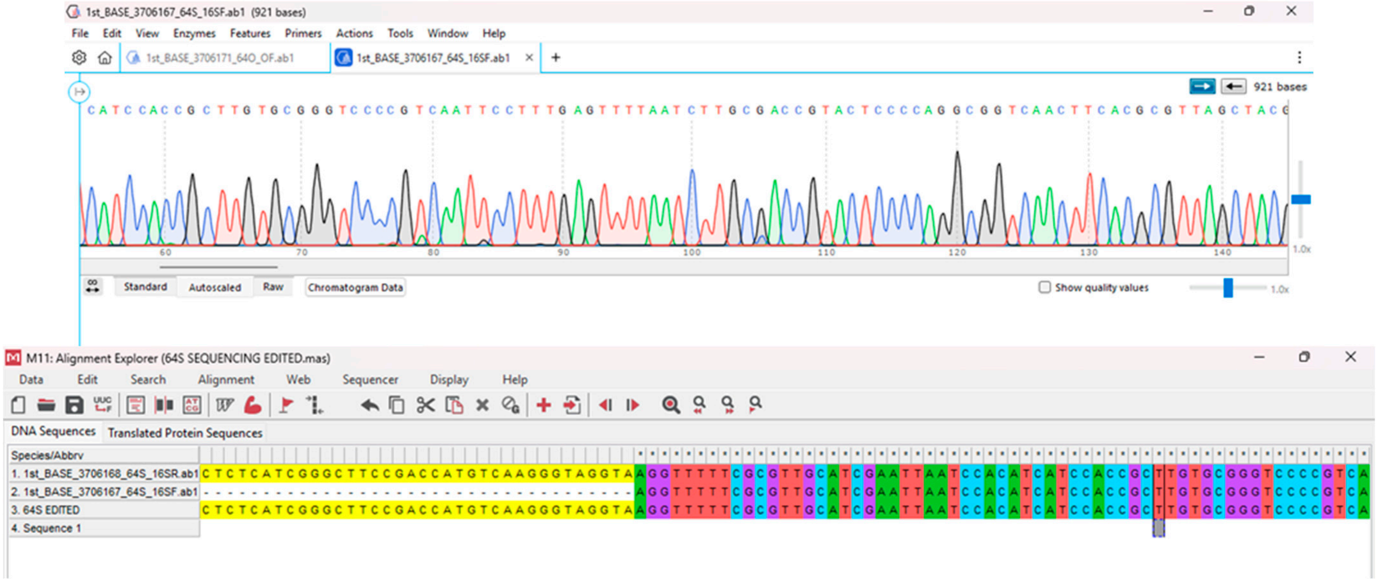

Figure S2. Alignment of the 16S sequencing result using MEGA X to complete the areas where the chromatogram quality was not good to obtain the final completed sequence of the 16S for the sample

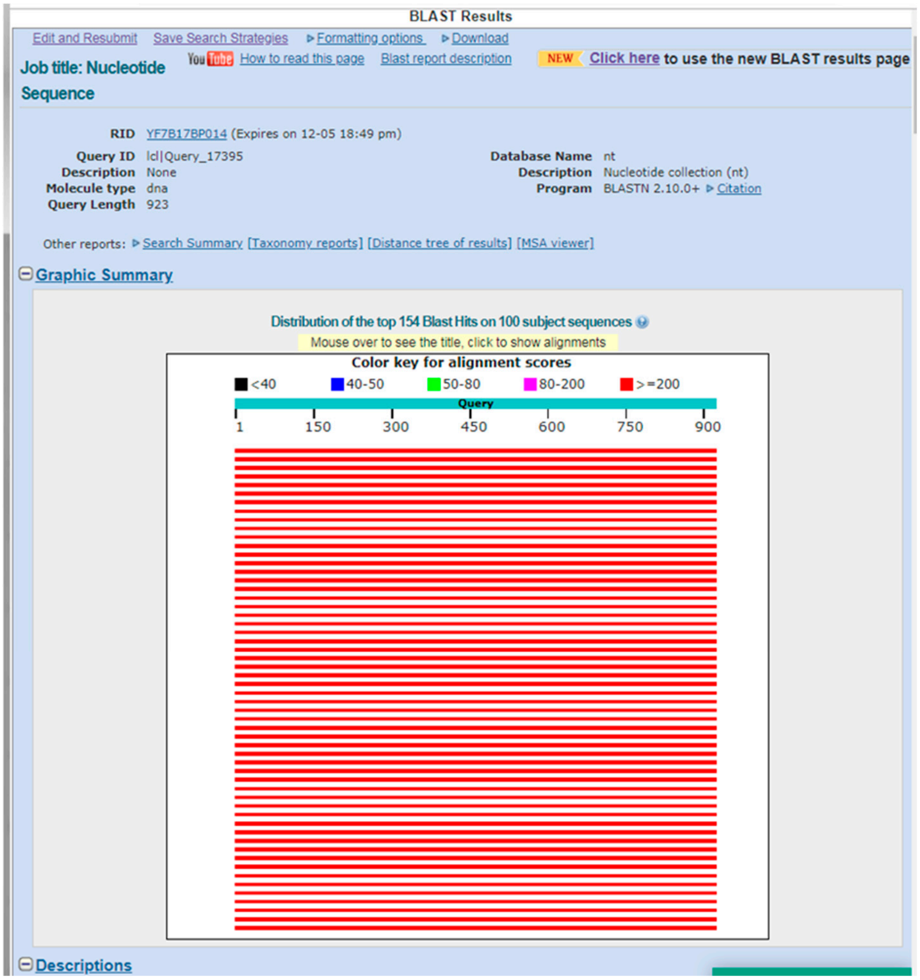

Figure S3. Chromatogram of the sequence result of orf2 gene from sample #64

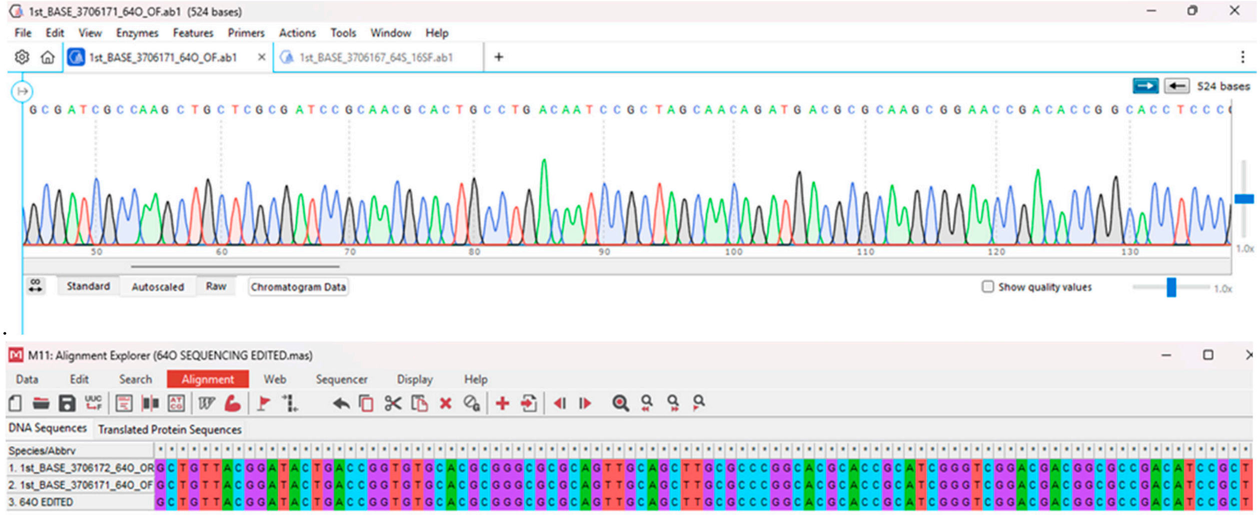

Figure S4. Alignment of the orf2 sequencing result using MEGA X to complete the areas where the chromatogram quality was not good to obtain the final completed sequence of the orf2 for the sample

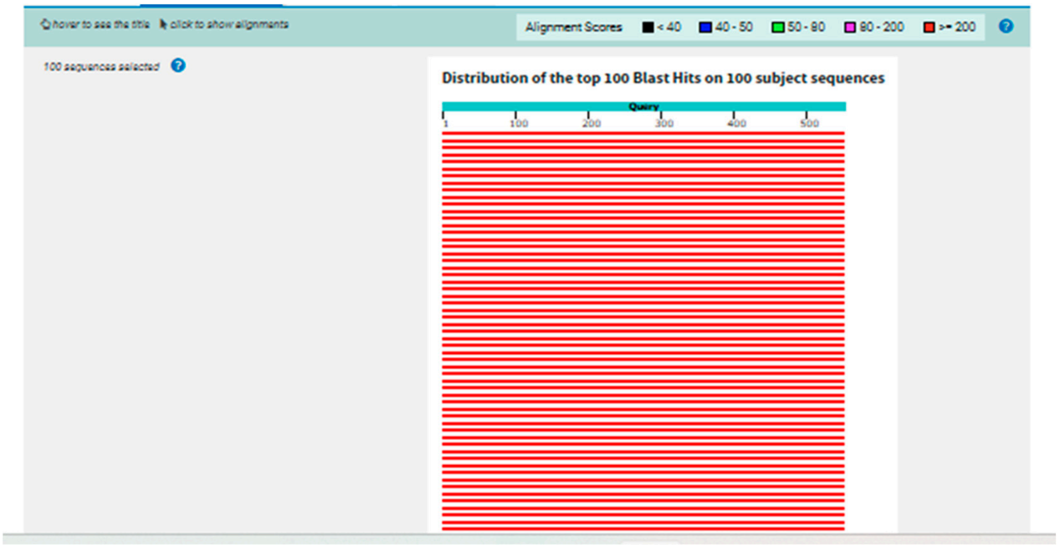

Supplement: Supplementary file 1 [file pathogens-15-00835-s001.zip › pathogens-4416810-supplementary.pdf]
